# A Universal Theoretical Framework in Material Characterization for Tailored Porous Surface Design

**DOI:** 10.1038/s41598-019-45350-5

**Published:** 2019-06-19

**Authors:** Muhammad Burhan, Muhammad Wakil Shahzad, Kim Choon Ng

**Affiliations:** King Abdullah University of Science and Technology, Thuwal, 23955-6900 Saudi Arabia

**Keywords:** Computational methods, Imaging techniques

## Abstract

The distinct interaction of adsorbate-adsorbent pair is attributed to the characteristics of heterogeneous surface and structure of porous materials. In material science, the porous structure is modified in response to certain applications. Backed by the chemical recipes, such conventional approach rely on the material characterization techniques to verify the resultant porous structure and its interaction with the adsorbate molecules. Such a practice is best assisted by a theoretical approach that can pre-define the required heterogeneous structure of porous surfaces and its role in selective adsorbate-adsorbent interaction, to facilitate material scientists for the synthesis of only those energy sites which can enhance or tailor its responses for a certain application or target. It has been reported here that the understanding of porous structure in terms of energy sites and their distribution, which controls the adsorbate-adsorbent interaction, is the key for porous surface engineering. Understanding of such porous surface characteristics empower the scientists to alter kinetics and thermodynamics of material according to the ‘sweet spots’ of an application. Therefore, a theoretical framework, to express the energy sites and their distribution over the porous heterogeneous surface, is demonstrated here as a prerequisite criterion for porous material development and characterization.

## Introduction

The development of porous adsorbent materials in relation to predefined characteristics, has both theoretical significance and practical importance^1–7^. Crafting these adsorbent materials for perfect pore and energy sites distribution is catching researcher’s interest. The synthesis of these structured sieves can be carried out either by innovative recipe for new materials^8^ or by the post treatment of the parent structures. Thus, the synthesized material selectively enhance the characteristics of adsorbent surface for gas/vapor uptake^9,10^. Physical adsorption test of gas molecules is then performed first to analyze the material response^11–13^. With such quick analysis, the material characteristics in terms of pore size, pore volume, active area and uptake capacity can be known^14–16^. Electron microscopy and X-ray diffraction are also used to supplement the topography details of the porous surface^17,18^. However, this practice does not highlight the change in the energy sites of the heterogeneous surface that resulted in or caused by such material properties.

It is important to mention here that each adsorbent-adsorbate pair has distinct interaction, depending upon the heterogeneity and the distribution of energy sites over the porous surface. However, the availability and activation of these adsorption sites, at certain energy level and adsorbate concentration, define the isotherm characteristics of adsorbent-adsorbate pair, for all six IUPAC types^19,20^. The current imaging and scanning techniques are not yet capable of capturing the true topography of porous surface and the resultant adsorption phenomena and that is why, the efforts are being made on theoretical model based visualization techniques^21^. With increasing interest and need of material synthesis, it is critical to understand the true topography of the parent material and the required change which can reflect the desired characteristics. The current ‘effect and cause’ approach must be replaced with the vice versa methodology. Therefore, for the first time, a generalized theoretical-graphical technique is developed, which not only provides visualization of the heterogeneous porous structure and insight to the adsorbent-adsorbate pair interaction, but a change in the topography of porous surface can be envisioned, ahead of synthesis, for the tailored material response.

## Result and Discussion

**Figure 1:**
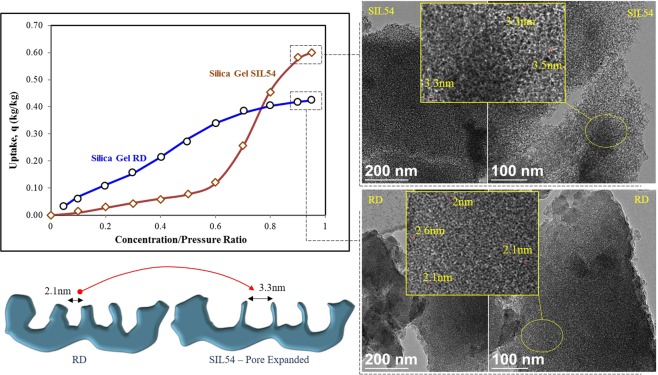
Effect of Pore and Surface Area Expansion on the Topography of Silica; Isotherm Data and TEM Images.

The effect of material synthesis on the topography of RD (Regular Density) silica, for pore expansion, is shown in Fig. 1. The synthesized material, SIL54 (micronized silica) showed increased porosity with expanded pore size (pore volume 0.8 ml/g, pore size 3.3 nm), as can be seen from TEM images at 100 nm scale bar. The RD silica (pore volume 0.35 ml/g, pore size 2.1 nm) has very stable and gradual uptake of water vapors till saturation at 0.42(kg/kg), reflecting very high surface heterogeneity. On the other hand, despite higher uptake, the heterogeneity of SIL54 is reduced as sharp adsorbate uptake can be observed after concentration ratio of 0.6. Such response is important for separation of water molecules from air, in humid climate regions, against the gradual uptake characteristics of conventional RD silica. Therefore, it is important to analyze and understand the structural and topographical difference of both materials.

The adsorption surface consists of groups of tiny pores with different energy levels. For the adsorbate molecule, to be attracted by the adsorbent pore, it must have critical energy level corresponding to that adsorption site energy i.e. ${\varepsilon }_{c}=-\,RT\,\mathrm{ln}\,Kp$. Therefore, the total adsorption uptake is the integral of individual uptake by adsorption energy sites with energy level corresponding to the critical energy of the adsorbate molecule i.e. ${\theta }_{t}={\int }_{{\varepsilon }_{c}}^{\infty }X(\varepsilon )d\varepsilon$. If availability of these group of energy sites is denoted by probability factor ‘α’ then the energy distribution function of the porous heterogeneous surface is proposed to be as.

$$\begin{array}{c}\begin{array}{ccc}X(\varepsilon )=\sum _{i=1}^{n}{\alpha }_{i}\,{\{\frac{\exp (\frac{{\rm{\Delta }}\varepsilon -{\varepsilon }_{oi}}{{m}_{i}})}{{m}_{i}{[1+\exp (\frac{{\rm{\Delta }}\varepsilon -{\varepsilon }_{oi}}{{m}_{i}})]}^{2}}\}}_{i} & {\rm{with}}\,{\rm{adsorption}}\,{\rm{uptake}}\,{\rm{as}} & {\theta }_{t}=\sum _{i=1}^{n}{\alpha }_{i}{\{\frac{{(\frac{p}{{p}_{s}}\exp (\frac{{\varepsilon }_{oi}}{RT}))}^{\frac{RT}{mi}}}{1+{(\frac{p}{{p}_{s}}\exp (\frac{{\varepsilon }_{oi}}{RT}))}^{\frac{RT}{mi}}}\}}_{i}\end{array}\end{array}$$

**Figure 2:**
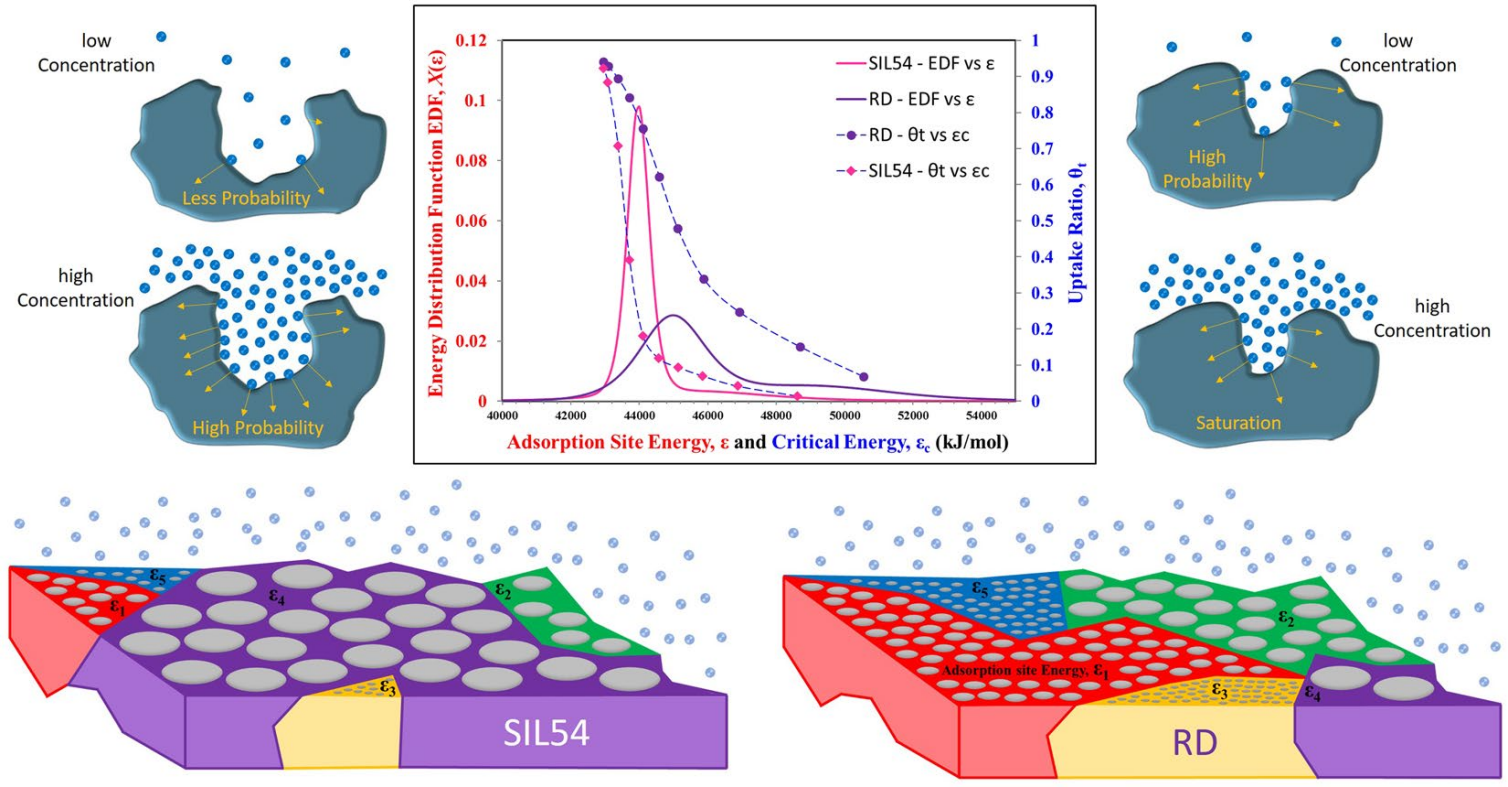
Adsorption Sites Energy Distribution of Silica (RD and SIL54) with Illustration of Resultant Topographical Change.

Based upon the parametric information of surface heterogeneity ‘m’, median energy level ‘ε_o_’ and the probability ‘*α’* of the availability of such group of energy sites, extracted by the proposed mathematical model through isotherms data of RD and SIL54, the topography of the both RD and SIL54, in form of energy sites distribution and surface uptake against critical energy level, is shown in Fig. 2. From energy distribution function (EDF) graph of RD silica, a highly heterogeneous surface can be observed with mean energy level of 45000 KJ/mol. Such high surface heterogeneity is illustrated in the lower part of Fig. 2 where most of the adsorption sites of energy value ε_1_, ε_2_, ε_3_ ….. ε_5_ have similar density or availability. On the other hand, pore expanded silica SIL54 showed less heterogeneity, resulting in sharp adsorption uptake, with the mean of energy sites being shifted to 44000 KJ/mol. This less surface heterogeneity is also illustrated in the lower part of Fig. 2 where density or availability of adsorption site of mean energy value ε_4_ is very high as compared to the other adsorption sites of energy value ε_1_, ε_2_, ε_3_ and ε_5_. In addition, the shift of mean site energy value is due to the pore expansion of conventional RD silica as the adsorption trend goes towards the high pressure side for large size pores. This is because of the fact that the probability of adsorbate molecules, to be close enough to be captured by the porous surface, becomes higher at high concentration. As a results, a large uptake is noticed only when the concentration of molecules increases. However, for small size pores, such probability is higher even at the low concentration as there is high chance of them being captured by the porous surface, as graphically explained in Fig. 2. However, for smaller pores, the high concentration/pressure of adsorbate molecules does not significantly affect the overall adsorption uptake but rather shows the saturation phenomena for that particular pore.

For the pore expanded silica SIL54, a drop in the critical energy level can be observed as larger pores require high concentration/pressure to respond i.e. ${\varepsilon }_{c}=-\,RT\,\mathrm{ln}\,Kp$. The variation of critical energy ‘ε_c_’ against uptake, Fig. 2, explains the response of porous surface in terms of adsorption sites availability. It gives insight to the rate of adsorption and the shape of isotherms as the adsorption can only happen when an adsorption site is available i.e. the energy site value is higher than the critical energy level. The rate of adsorption relies on the gradient of EDF and its total occurrence or probability over the porous surface, after its availability. That is why, a sudden rise in adsorption uptake can be noticed with the availability of median energy site. However, it is also important to note that the overall higher uptake of SIL54 is not due to higher probability of median energy site, but rather is because of larger pore volume. On one hand, if larger pores requires high concentration/pressure to respond and yet, these low energy sites of larger pores are easy to regenerate. However, for smaller pores, very low concentration/pressure levels are required for the regeneration of porous surface.

**Figure 3:**
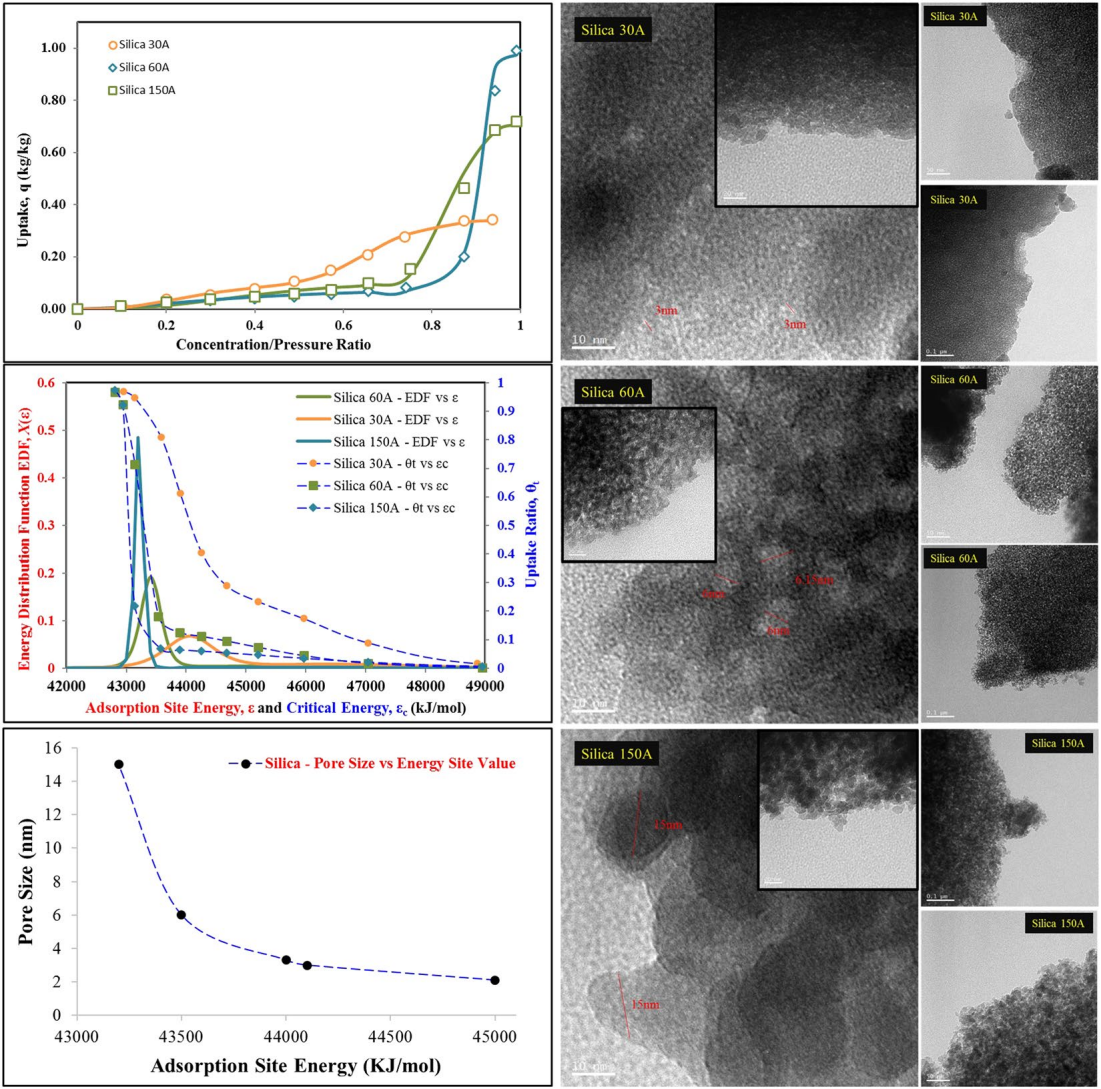
Relationship of Pore Size with Mean Adsorption Energy Site for Tailored Response of the Material.

In order to validate the applicability and generality of the proposed theoretical framework, three additional samples of synthesized silica of pore sizes 30 Å (0.4 ml/g), 60 Å (0.75 ml/g) and 150 Å (1.15 ml/g), are considered and their isotherm data and surface topography, in form of EDF and critical energy ‘ε_c_’, are examined in Fig. 3. For 30 Å silica, owing to similar pore size and heterogeneity, alike topography and uptake response has been observed as that of SIL54. The pore expansion is only causing some of the smaller pores to expand to the mean pore size. In addition, the rate of adsorption is observed to be increasing after a concentration ratio higher than 0.6. However, its total adsorption uptake is lower due to smaller pore volume which is because of smaller density or total number of pores, as compared to SIL54.

The most important aspect in the application of porous materials, is their response at specific concentration, which depends upon the mean site energy of the porous surface, as explained before. It can be noticed that the mean energy site value has shifted from 44100 kJ/mol to 43200 kJ/mol for these three silica samples when the mean pore size is expanded from 3 nm to 15 nm. In can also be seen that with the pore expansion, the surface heterogeneity of the materials reduces drastically. In order to correlate and understand the relationship between topography of the porous silica material and its energy sites or concentrations response, the spread of mean energy site value against pore size, is shown in Fig. 3. For analysis, it can be noticed that the change in mean energy site value per pore size is higher for the smaller pores i.e. 6nm-2nm. After this pore size limit, the change in mean energy site value is minute for larger pores. This reflects the saturation of silica adsorption energy sites as its availability is increasing from 3% to 50% for pore expansion 2.1 nm to 15 nm.

**Figure 4:**
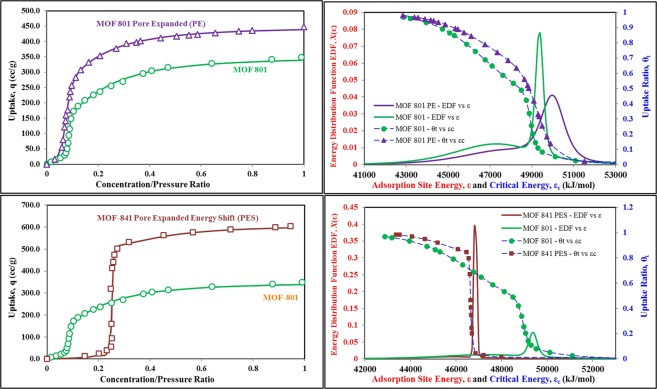
The isotherm data^22^ and the extracted topographical information for MOF 801, MOF 801-PE and MOF 841-PES.

The proposed theoretical-graphical technique is now applied to material organic framework (MOF 801) case, to visualize the effects on the topography of its heterogeneous surface for two synthesized samples i.e. pore expansion without energy shift (PE) and pore expansion with energy shift (PES). The isotherm data^22^ and the extracted topographical information for MOF 801 and its two synthesized variants MOF 801-PE and MOF 841-PES, are shown in Fig. 4. The synthesized MOF 801-PE is a case where it is targeted for higher uptake, without any significant energy shift. However, the heterogeneity of energy sites and so as pores, is reduced due expansion of smaller pores, with energy level of 50000 KJ/mol or higher, to the mean energy level. As a result, overall higher uptake is observed due to increase in pore volume, from 0.45 ml/g of MOF 801-PE to 0.27 ml/g of MOF-801. On the other hand, for MOF 841-PES, a significant shift in mean energy level is observed with drastic reduction in surface heterogeneity. With pore expansion and high availability of large mean pore size, high pore volume of 0.53 ml/g is observed, which resulted in overall highest uptake of 600 cc/g. In addition, the response of the material is also shifted from concentration/pressure ratio of 0.1 to 0.25.

## Conclusion

Owing to inadequacy of conventional imaging techniques, the universal theoretical framework has been graphically demonstrated to visualize and understand the porous surface topography. This approach has the potential to guide and conduct tailored synthesis of porous materials. By linking the relation between the porous surface topography and the adsorption energy site, complete quantification of the porous heterogeneous surface can be accurately made for custom needs of uptake or concentration responses in the separation, purification and storage applications.

## Materials and Methods

### Universal theoretical framework

The coverage or total adsorption uptake of porous surface can be simplified and expressed by the integration of localized uptake of quasi-static patches or energy sites i.e.

1 $$\begin{array}{ccc}{\theta }_{t}=\mathop{{\int }_{0}^{\infty }\{\theta (\varepsilon )X(\varepsilon )\}d\varepsilon }\limits_{\mathop{\longleftrightarrow }\limits_{all\,sites}} & \mathop{\mathrm{lim}}\limits_{T\to 0}\theta (\varepsilon )={\theta }_{c}(\varepsilon )=\{\begin{array}{c}\begin{array}{c}0\,for\,{\rm{\Delta }}\varepsilon \le {\varepsilon }_{c}\\ 1\,for\,{\rm{\Delta }}\varepsilon \ge {\varepsilon }_{c}\end{array}\\ {\varepsilon }_{c}=-\,RT\,\mathrm{ln}\,Kp\end{array} & {\theta }_{t}={\int }_{{\varepsilon }_{c}}^{\infty }X(\varepsilon )d\varepsilon \end{array}$$

where ‘*θ(ε)*’ represents localized adsorption uptake, ‘*X(ε)*’ the energy distribution function, ‘*ε*_*c*_’ critical energy level of adsorbate molecule and ‘${\rm{\Delta }}\varepsilon ={\varepsilon }_{d}-{\varepsilon }_{a}=\varepsilon -{h}_{fg}$’ the difference between adsorption and desorption energy or the difference between adsorption energy site and the vaporization energy.

With condensation approximation (CA) applied, it can be seen that the energy distribution function of adsorption energy sites, *X(ε)* is the only variable that can define the total adsorption uptake (*θ*_*t*_). Therefore, the accurate forecasting of adsorption sites distribution is the key here to truly capture the adsorption uptake. An energy distribution function in symmetrical Gaussian function form, is introduced here with embedded probability factor *α*, to meaningfully express the characteristics of the adsorption energy sites and their distribution over the heterogeneous surface i.e.

2 $$X(\varepsilon )=\sum _{i=1}^{n}{\alpha }_{i}{\{\frac{\exp (\frac{{\rm{\Delta }}\varepsilon -{\varepsilon }_{oi}}{{m}_{i}})}{{m}_{i}{[1+\exp (\frac{{\rm{\Delta }}\varepsilon -{\varepsilon }_{oi}}{{m}_{i}})]}^{2}}\}}_{i}$$

Where the sum of probability factor $\sum _{i=1}^{n}{\alpha }_{i}$ equal to ‘1’ i.e. ${\alpha }_{1}+{\alpha }_{2}=1$ ‘OR’ $\alpha +(1-\alpha )=1$

$$\begin{array}{c}X(\varepsilon )={\alpha }_{1}\frac{\exp (\frac{{\rm{\Delta }}\varepsilon -{\varepsilon }_{o1}}{{m}_{1}})}{{m}_{1}{[1+\exp (\frac{{\rm{\Delta }}\varepsilon -{\varepsilon }_{o1}}{{m}_{1}})]}^{2}}+{\alpha }_{2}\frac{\exp (\frac{{\rm{\Delta }}\varepsilon -{\varepsilon }_{o2}}{{m}_{2}})}{{m}_{2}{[1+\exp (\frac{{\rm{\Delta }}\varepsilon -{\varepsilon }_{o2}}{{m}_{2}})]}^{2}}\\ ^{\prime} {\rm{OR}}^{\prime} \\ X(\varepsilon )=\alpha \frac{\exp (\frac{{\rm{\Delta }}\varepsilon -{\varepsilon }_{o1}}{{m}_{1}})}{{m}_{1}{[1+\exp (\frac{{\rm{\Delta }}\varepsilon -{\varepsilon }_{o1}}{{m}_{1}})]}^{2}}+(1-\alpha )\frac{\exp (\frac{{\rm{\Delta }}\varepsilon -{\varepsilon }_{o2}}{{m}_{2}})}{{m}_{2}{[1+\exp (\frac{{\rm{\Delta }}\varepsilon -{\varepsilon }_{o2}}{{m}_{2}})]}^{2}}\end{array}$$

By integrating the introduced energy distribution function eq. (2) in total uptake expression eq. (1), for available adsorption sites over the critical energy level’${\varepsilon }_{c}$’:

3 $${\theta }_{t}={\int }_{{\varepsilon }_{c}}^{\infty }[\sum _{i=1}^{n}{\alpha }_{i}{\{\frac{\exp (\frac{{\rm{\Delta }}\varepsilon -{\varepsilon }_{o}}{m})}{m{[1+\exp (\frac{{\rm{\Delta }}\varepsilon -{\varepsilon }_{o}}{m})]}^{2}}\}}_{i}]d\varepsilon $$

4 $${\theta }_{t}={\int }_{{\varepsilon }_{c}}^{\infty }\{\alpha \frac{\exp (\frac{{\rm{\Delta }}\varepsilon -{\varepsilon }_{o1}}{{m}_{1}})}{{m}_{1}{[1+\exp (\frac{{\rm{\Delta }}\varepsilon -{\varepsilon }_{o1}}{{m}_{1}})]}^{2}}+(1-\alpha )\frac{\exp (\frac{{\rm{\Delta }}\varepsilon -{\varepsilon }_{o2}}{{m}_{2}})}{{m}_{2}{[1+\exp (\frac{{\rm{\Delta }}\varepsilon -{\varepsilon }_{o2}}{{m}_{2}})]}^{2}}\}d\varepsilon $$

By solving integral,

5 $${\theta }_{t}=\alpha {[1+\exp (\frac{{\varepsilon }_{c}-{\varepsilon }_{o1}}{{m}_{1}})]}^{-1}+(1-\alpha ){[1+\exp (\frac{{\varepsilon }_{c}-{\varepsilon }_{o2}}{{m}_{2}})]}^{-1}$$

However, $${\varepsilon }_{c}=-RT\,\mathrm{ln}\,Kp$$

6 $${\theta }_{t}=\alpha {[1+\exp (\frac{-RT\mathrm{ln}Kp-{\varepsilon }_{o1}}{{m}_{1}})]}^{-1}+(1-\alpha ){[1+\exp (\frac{-RT\mathrm{ln}Kp-{\varepsilon }_{o2}}{{m}_{2}})]}^{-1}$$

By simplifying further, we can get:

$$\begin{array}{c}{\theta }_{t}=\alpha {[1+\exp (\frac{-RT\mathrm{ln}Kp}{{m}_{1}})\exp (\frac{-{\varepsilon }_{o1}}{{m}_{1}})]}^{-1}+(1-\alpha ){[1+\exp (\frac{-RT\mathrm{ln}Kp}{{m}_{2}})\exp (\frac{-{\varepsilon }_{o2}}{{m}_{2}})]}^{-1}\\ {\theta }_{t}=\alpha {[1+\exp (\mathrm{ln}K{p}^{\frac{-RT}{{m}_{1}}})\exp (\frac{-{\varepsilon }_{o1}}{{m}_{1}})]}^{-1}+(1-\alpha ){[1+\exp (\mathrm{ln}K{p}^{\frac{-RT}{{m}_{2}}})\exp (\frac{-{\varepsilon }_{o2}}{{m}_{2}})]}^{-1}\\ {\theta }_{t}=\alpha {[1+K{p}^{\frac{-RT}{{m}_{1}}}\exp {(\frac{{\varepsilon }_{o1}}{RT})}^{\frac{-RT}{{m}_{1}}}]}^{-1}+(1-\alpha ){[1+K{p}^{\frac{-RT}{{m}_{2}}}\exp {(\frac{{\varepsilon }_{o2}}{RT})}^{\frac{-RT}{{m}_{2}}}]}^{-1}\\ {\theta }_{t}=\alpha {[1+\frac{1}{{(Kp\exp (\frac{{\varepsilon }_{o1}}{RT}))}^{\frac{RT}{m1}}}]}^{-1}+(1-\alpha ){[1+\frac{1}{{(Kp\exp (\frac{{\varepsilon }_{o2}}{RT}))}^{\frac{RT}{m2}}}]}^{-1}\\ {\theta }_{t}=\alpha [\frac{{(Kp\exp (\frac{{\varepsilon }_{o1}}{RT}))}^{\frac{RT}{m1}}}{1+{(Kp\exp (\frac{{\varepsilon }_{o1}}{RT}))}^{\frac{RT}{m1}}}]+(1-\alpha )[\frac{{(Kp\exp (\frac{{\varepsilon }_{o2}}{kT}))}^{\frac{RT}{m2}}}{1+{(Kp\exp (\frac{{\varepsilon }_{o2}}{RT}))}^{\frac{RT}{m2}}}]\end{array}$$

The universal model can be obtained by letting the adsorption equilibrium constant $K=1/{p}_{s}$ i.e.

7 $${\theta }_{t}=\alpha [\frac{{(\frac{p}{{p}_{s}}\exp (\frac{{\varepsilon }_{o1}}{RT}))}^{\frac{RT}{m1}}}{1+{(\frac{p}{{p}_{s}}\exp (\frac{{\varepsilon }_{o1}}{RT}))}^{\frac{RT}{m1}}}]+(1-\alpha )[\frac{{(\frac{p}{{p}_{s}}\exp (\frac{{\varepsilon }_{o2}}{kT}))}^{\frac{RT}{m2}}}{1+{(\frac{p}{{p}_{s}}\exp (\frac{{\varepsilon }_{o2}}{RT}))}^{\frac{RT}{m2}}}]$$

8 $${\theta }_{t}=\sum _{i=1}^{n}{\alpha }_{i}{\{\frac{{(\frac{p}{{p}_{s}}\exp (\frac{{\varepsilon }_{oi}}{RT}))}^{\frac{RT}{mi}}}{1+{(\frac{p}{{p}_{s}}\exp (\frac{{\varepsilon }_{oi}}{RT}))}^{\frac{RT}{mi}}}\}}_{i}$$

### TEM experiment

The TEM images were obtained using Philips 420 TEM equipment which was operated in the bright field imaging mode at 120 kV. The magnification levels used for reported TEM images were 210,000X, 96,000X, 47,000X and 28,000X. Each powder sample was deposited onto TEM support grid which was then imaged immediately using TEM. The images were recorded at random locations from electron transparent regions.

## Supplementary information

Supplementary Information

## Data Availability

All data generated or analyzed during this study are included in this published article (and its Supplementary Information).
