# Supplementary material for: A Universal Theoretical Framework in Material Characterization for Tailored Porous Surface Design
Source: Sci Rep. 2019 Jun 19;9:8773. doi: 10.1038/s41598-019-45350-5 (PMC6584588; doi:10.1038/s41598-019-45350-5)
Supplement: Supplementary file 1 — Supplementary Information [file 41598_2019_45350_MOESM1_ESM.docx]

**A Universal Theoretical Approach in Material Characterization for Tailored Porous Surface Design**

^1^Muhammad Burhan*, ^2^Muhammad Wakil Shahzad, ^3^Kim Choon Ng

^1-3^King Abdullah University of Science and Technology, Thuwal 23955-6900, Saudi Arabia.

Emails: ^1^[muhammad.burhan@kaust.edu.sa](mailto:muhammad.burhan@kaust.edu.sa); ^2^[muhammad.shahzad@kaust.edu.sa](mailto:muhammad.shahzad@kaust.edu.sa); ^3^[kimchoon.ng@kaust.edu.sa](mailto:kimchoon.ng@kaust.edu.sa)

**Supplementary Information:**

The parameters related to presented adsorption isotherm predictions and corresponding EDF are given in the tables below:

Table 1: Key values of parameters of universal adsorption model for Silica-RD isotherm

| **Parameter** | **Value** | **Parameter** | **Value** |
| --- | --- | --- | --- |
| ε_o1_ (J/mol) | 5799.9 | ε_o2_ (J/mol) | 1729.6 |
| m1 (J/mol) | 1529.9 | m2 (J/mol) | 635.2 |
| α_1_ | 0.307 | α_2_ | 0.693 |
| P_s_ (kPa) | 4.247 | q*(kg/kg) | 0.45 |

Table 2: Key values of parameters of universal adsorption model for Silica-SIL54 isotherm

| **Parameter** | **Value** | **Parameter** | **Value** |
| --- | --- | --- | --- |
| ε_o1_ (J/mol) | 732.8 | ε_o2_ (J/mol) | 2314.9 |
| m1 (J/mol) | 211.7 | m2 (J/mol) | 1401.7 |
| α_1_ | 0.8128 | α_2_ | 0.1872 |
| P_s_ (kPa) | 4.247 | q*(kg/kg) | 0.65 |

Table 3: Key values of parameters of universal adsorption model for Silica-30Å isotherm

| **Parameter** | **Value** | **Parameter** | **Value** |
| --- | --- | --- | --- |
| ε_o1_ (J/mol) | 1093.5 | ε_o2_ (J/mol) | 3456.7 |
| m1 (J/mol) | 266.8 | m2 (J/mol) | 917.06 |
| α_1_ | 0.6965 | α_2_ | 0.3035 |
| P_s_ (kPa) | 9.595 | q*(kg/kg) | 0.35 |

Table 4: Key values of parameters of universal adsorption model for Silica-60Å isotherm

| **Parameter** | **Value** | **Parameter** | **Value** |
| --- | --- | --- | --- |
| ε_o1_ (J/mol) | 444.3 | ε_o2_ (J/mol) | 2216.01 |
| m1 (J/mol) | 110 | m2 (J/mol) | 977 |
| α_1_ | 0.8356 | α_2_ | 0.1644 |
| P_s_ (kPa) | 9.595 | q*(kg/kg) | 0.735 |

Table 5: Key values of parameters of universal adsorption model for Silica-150Å isotherm

| **Parameter** | **Value** | **Parameter** | **Value** |
| --- | --- | --- | --- |
| ε_o1_ (J/mol) | 283.3 | ε_o2_ (J/mol) | 2545.3 |
| m1 (J/mol) | 45.92 | m2 (J/mol) | 1492.7 |
| α_1_ | 0.9099 | α_2_ | 0.0901 |
| P_s_ (kPa) | 9.595 | q*(kg/kg) | 0.992 |

Table 6: Key values of parameters of universal adsorption model for MOF 801 isotherm

| **Parameter** | **Value** | **Parameter** | **Value** |
| --- | --- | --- | --- |
| ε_o1_ (J/mol) | 4023.0 | ε_o2_ (J/mol) | 6077.1 |
| m1 (J/mol) | 1330.9 | m2 (J/mol) | 121.9 |
| α_1_ | 0.6494 | α_2_ | 0.3506 |
| P_s_ (kPa) | 3.17 | q*(cc/g) | 349 |

Table 7: Key values of parameters of universal adsorption model for MOF 801-PE isotherm

| **Parameter** | **Value** | **Parameter** | **Value** |
| --- | --- | --- | --- |
| ε_o1_ (J/mol) | 4331.1 | ε_o2_ (J/mol) | 6430.8 |
| m1 (J/mol) | 1399.2 | m2 (J/mol) | 355.13 |
| α_1_ | 0.4911 | α_2_ | 0.5089 |
| P_s_ (kPa) | 3.17 | q*(cc/g) | 448 |

Table 8: Key values of parameters of universal adsorption model for MOF 841-PES isotherm

| **Parameter** | **Value** | **Parameter** | **Value** |
| --- | --- | --- | --- |
| ε_o1_ (J/mol) | 2413.9 | ε_o2_ (J/mol) | 3410.6 |
| m1 (J/mol) | 866.21 | m2 (J/mol) | 32.47 |
| α_1_ | 0.212 | α_2_ | 0.788 |
| P_s_ (kPa) | 3.17 | q*(cc/g) | 606 |
